# Renal interstitial fibrotic assessment using non-Gaussian diffusion kurtosis imaging in a rat model of hyperuricemia

**DOI:** 10.1186/s12880-024-01259-8

**Published:** 2024-04-03

**Authors:** Ping-Kang Chen, Zhong-Yuan Cheng, Ya-Lin Wang, Bao-Jun Xu, Zong-Chao Yu, Zhao-Xia Li, Shang-Ao Gong, Feng-Tao Zhang, Long Qian, Wei Cui, You-Zhen Feng, Xiang-Ran Cai

**Affiliations:** 1 Medical Imaging Center, The First Affiliated Hospital of Jinan University, No.613 West Huangpu Avenue, Tianhe District, Guangzhou, Guangdong 510630 China; 2 Nephrology department, The First Affiliated Hospital of Jinan University, Guangzhou, Guangdong China; 3 Department of Rheumatology, The First Affiliated Hospital of Jinan University, Guangzhou, Guangdong China; 4 Intervention department, Huazhong University of Science and Technology Union Shenzhen Hospital, Shenzhen, Guangdong China; 5 Department of Biomedical Engineering, College of Engineering, Peking University, Beijing, China; 6 MRI Research, GE Healthcare, Beijing, China

**Keywords:** Hyperuricemia, Diffusion kurtosis imaging, Magnetic resonance imaging, Renal interstitial fibrosis

## Abstract

**Background:**

To investigate the feasibility of Diffusion Kurtosis Imaging (DKI) in assessing renal interstitial fibrosis induced by hyperuricemia.

**Methods:**

A hyperuricemia rat model was established, and the rats were randomly split into the hyperuricemia (HUA), allopurinol (AP), and AP + empagliflozin (AP + EM) groups (*n* = 19 per group). Also, the normal rats were selected as controls (CON, *n* = 19). DKI was performed before treatment (baseline) and on days 1, 3, 5, 7, and 9 days after treatment. The DKI indicators, including mean kurtosis (MK), fractional anisotropy (FA), and mean diffusivity (MD) of the cortex (CO), outer stripe of the outer medulla (OS), and inner stripe of the outer medulla (IS) were acquired. Additionally, hematoxylin and eosin (H&E) staining, Masson trichrome staining, and nuclear factor kappa B (NF-κB) immunostaining were used to reveal renal histopathological changes at baseline, 1, 5, and 9 days after treatment.

**Results:**

The HUA, AP, and AP + EM group MK_OS_ and MK_IS_ values gradually increased during this study. The HUA group exhibited the highest MK value in outer medulla. Except for the CON group, all the groups showed a decreasing trend in the FA and MD values of outer medulla. The HUA group exhibited the lowest FA and MD values. The MK_OS_ and MK_IS_ values were positively correlated with Masson’s trichrome staining results (*r* = 0.687,* P* < 0.001 and* r* = 0.604,* P* = 0.001, respectively). The MD_OS_ and FA_IS_ were negatively correlated with Masson’s trichrome staining (*r* = -626,* P* < 0.0014 and* r* = -0.468,* P* = 0.01, respectively).

**Conclusion:**

DKI may be a non-invasive method for monitoring renal interstitial fibrosis induced by hyperuricemia.

**Supplementary Information:**

The online version contains supplementary material available at 10.1186/s12880-024-01259-8.

## Background

Hyperuricemia (HUA) is a metabolic disease that results from increased production and/or reduced excretion of serum uric acid (SUA) [1]. The prevalence of both HUA and gout has increased worldwide, making these major public health problems [2, 3]. Hyperuricemia is closely associated with renal injury [4]. High SUA levels stimulate the nuclear factor kappa B (NF-κB) signaling pathway and releasing proinflammatory cytokines [5, 6]. These factors induce the infiltration of inflammatory cells, trigger renal tubule injury, and contribute to renal fibrosis [7–9].

Currently, urate-lowering therapy (ULT) is the standard therapy for patients with hyperuricemia and gout [10, 11]. Allopurinol (AP) is a xanthine oxidase inhibitor that is commonly used in ULT. In some studies, AP inhibits the NOD-like receptor thermal protein domain associated protein 3 (NLRP3)/NF-κB inflammasome activation by lowering uric acid [12, 13] and decreasing renal fibrosis [14, 15]. Sodium-dependent glucose transporter 2 inhibitors (SGLT2i), like canagliflozin, dapagliflozin, and empagliflozin are a new class of antidiabetic drugs. In recent years, some studies have indicated that SGLT2i reduce SUA levels while regulating blood sugar levels [16, 17].

Declining kidney function is closely associated with renal interstitial fibrosis [18, 19]. Therefore, early, and accurate detection of interstitial fibrosis related to HUA is crucial for both renal function evaluation and treatment selection. Currently, renal biopsy is the gold standard for detecting renal fibrosis. However, since it is invasive, painful, and accompanied by complications, it is not routinely used to monitor the progression of renal fibrosis [20]. Therefore, a non-invasive method to accurately diagnose hyperuricemia-induced kidney fibrosis is urgently needed.

In recent years, diffusion kurtosis imaging (DKI) is a promising magnetic resonance imaging technique which has been successfully applied to kidney disease such as IgA nephropathy [19], diabetic nephropathy [21], unilateral ureteral obstruction [18], and chronic kidney disease (CKD) [22]. Several studies have demonstrated that DKI is helpful in evaluating renal function changes and suggested that it may be used as a non-invasive tool to quantitatively assess renal function. Thus, in this study, we investigated the feasibility of using DKI to evaluate the relationship between kidney fibrosis and hyperuricemia alterations in renal fibrosis after urate-lowering therapy (ULT) were also analyzed using DKI.

## Methods

### Animal model and study design

This study was approved by the Ethics Committee of our university and conducted according to the Institutional Guidelines of Experimental Animal Care and Use. Seventy-eight male Sprague–Dawley rats (8 weeks, 200 ± 20 g) with license numbers of SCXK (Jing 20190008) purchased from Beijing HFK Bioscience Company were used in this study. After a week-long acclimatization period, all the rats were randomly divided into two groups: a control (CON) (*n* = 19) and an experimental (*n* = 59) group. Rats in the experimental group received potassium oxonate (Sigma,250 mg/kg, dissolved in normal saline) by intraperitoneal injection once a day and were fed a high-protein feed (containing 10% yeast extract). Control rats received the same volume of normal saline by intraperitoneal injection and were fed normal feed. After a week administration, uric acid level was test from tail vein samples. Animals in the experimental group with serum uric acid level two times more than control ones were included in this study [23] and subdivided HUA, allopurinol (AP), and AP + empagliflozin (AP + EM) groups. The HUA group served as the experimental group. The AP group received HUA treatment combined with allopurinol (150 mg/L) dissolved in their drinking water. The AP + EM group received HUA treatment in combination with allopurinol in drinking water intraperitoneally injection with empagliflozin (Sigma, 10 mg/kg) once a day.

Before treatment (baseline) and on days 1, 5, and 9 after treatment, three rats were randomly chosen from each group and euthanized. Both kidneys were harvested for pathological examination. Blood samples were collected from the abdominal aorta for analysis of laboratory parameters, including SUA, serum creatinine (Scr), and blood urea nitrogen (BUN) levels.

### MRI measurements

All MRI scans used a 3.0 T MRI scanner (Discovery 750, GE Healthcare, Milwaukee, USA) with an HD wrist array upper coil. MRI evaluation at baseline and on days 1, 3, 5, 7, and 9 after treatment. Six rats from each group were selected for the MRI assessment. Before MRI evaluation, the rats were received an intraperitoneal injection of 0.3% sodium pentobarbital (2 ml/kg) to maintain anesthesia during scanning. The DKI sequence was acquired in the coronal plane with three different *b*-values (0, 400, and 800 s/mm^2^) in 25 diffusion directions. The specific parameters of DKI were as follows: TR = 2000.0 ms, TE = 78.1 ms, slice thickness = 2.9 mm, gap = 0.1 mm, number of slices = 8, field of view = 8.0 × 4.0 cm, matrix = 128 × 96, NEX = 4.0, bandwidth = 166.7 kHz. The total acquisition time for DKI was 6 min and 50 s. The equation described by Jensen et al. [24] was used to calculate the DKI metrics:

$${\text{ln}}\left[{\text{S}}\left(n,b\right)/{{\text{S}}}_{0}\right]=b\sum\limits_{{i}=1}^{3}\sum\limits_{j=1}^{3}{n}_{i}{n}_{j}{D}_{{i}j }+\frac{1}{6}{b}^{2}{\overline{D} }^{2}\sum\limits_{{i}=1}^{3}\sum\limits_{j=1}^{3}\sum\limits_{k=1}^{3}\sum\limits_{l=1}^{3}{n}_{{i}}{n}_{j}{n}_{k}{n}_{l}{W}_{{i}jkl}$$

The diffusion signal intensity for the diffusion weighting *b* and diffusion-encoding direction *n* is represented by ${\text{S}}\left(n,b\right),$ ${{\text{S}}}_{0}$ is the signal intensity of ${b}_{0}$, and ${D}_{{i}j}$ is the diffusion tensor, and the kurtosis tensor is ${W}_{{i}jkl}$.

**Fig. 1:**
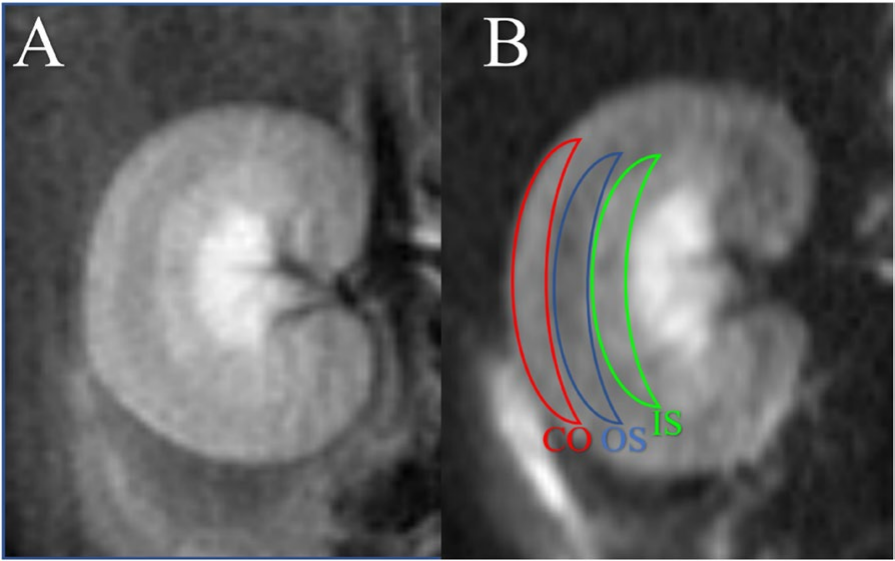
**A** T2 weighted image of kidney; **B** Regions of interest (ROI) into b = 0 s/mm^2^ image. From outside to inside: *CO*, cortex; *OS*, outer stripe of the outer medulla; and *IS*, inner stripe of the outer medulla

Two radiologists (with 4- and 5-years of imaging diagnostic experience) independently used a workstation (GE 4.5) to measure DKI data without using any constraints or smoothing in this study. According to previous studies [25, 26], the regions of interests (ROIs) were manually drawn on the right renal cortex (CO), outer stripe of the outer medulla (OS), and inner stripe of the outer medulla (IS) on the DKI (*b* = 0) images (Fig. 1). Three continuous coronal images were chosen to draw the ROIs, and the average values of the DKI parameters including mean kurtosis (MK), fractional anisotropy (FA), and mean diffusivity (MD) were calculated.

### Pathological and laboratory analysis

The kidneys were removed and fixed in a 10% neutral formalin solution for histological examination. Tissue specimens were cut into 4 μm slices after paraffin embedding and used for hematoxylin and eosin (H&E) staining, NF-κB immunostaining, and Masson trichrome staining. Renal interstitial fibrosis was evaluated by Masson’s trichrome staining. NF-κB immunostaining was conducted for assess the degree of NF-κB expression by using an NF-κB antibody (Bioss, Beijing, China). Image-Pro Plus software (version 6.0; Media Cybernetics, MD, USA) was used for semi-quantitative analysis of the mean optical density (MOD) of Masson’s trichrome staining and NF-κB immunostaining.

### Statistical analysis

Statistical analyses were performed using SPSS software (version 26.0; Chicago, IL, USA) and graphs were generated using GraphPad Prism software (version 8.0; IBM Corp., Armonk, NY, USA). All values were represented as the mean ± standard deviation (M ± SD) and *P* < 0.05 was considered statistically significant. The reproducibility of the DKI parameters between the two radiologists was assessed by the intraclass correlation coefficient (ICC) performed on the HUA group, and an ICC > 0.800 was considered consistent. One-way analysis of variance (ANOVA) was used to determine the differences among the four groups at the same time points as the DKI metrics. The Least—Significant Difference (LSD) test was employed as a post-hoc test for further comparison between groups. The histological analysis results and laboratory parameters between different groups at different time points were consistent with MRI scanning. Pearson’s correlation analysis was used to evaluate the correlation between DKI parameters and the results of Masson’s trichrome staining.

## Results

### Animal

In total, six rats were excluded from the study: four due to SUA levels below two times than those of controls, one due to anesthesia issues, and one due to poor image quality. Finally, 72 rats were included in this study.

### Comparison of DKI parameters between groups

**Fig. 2:**
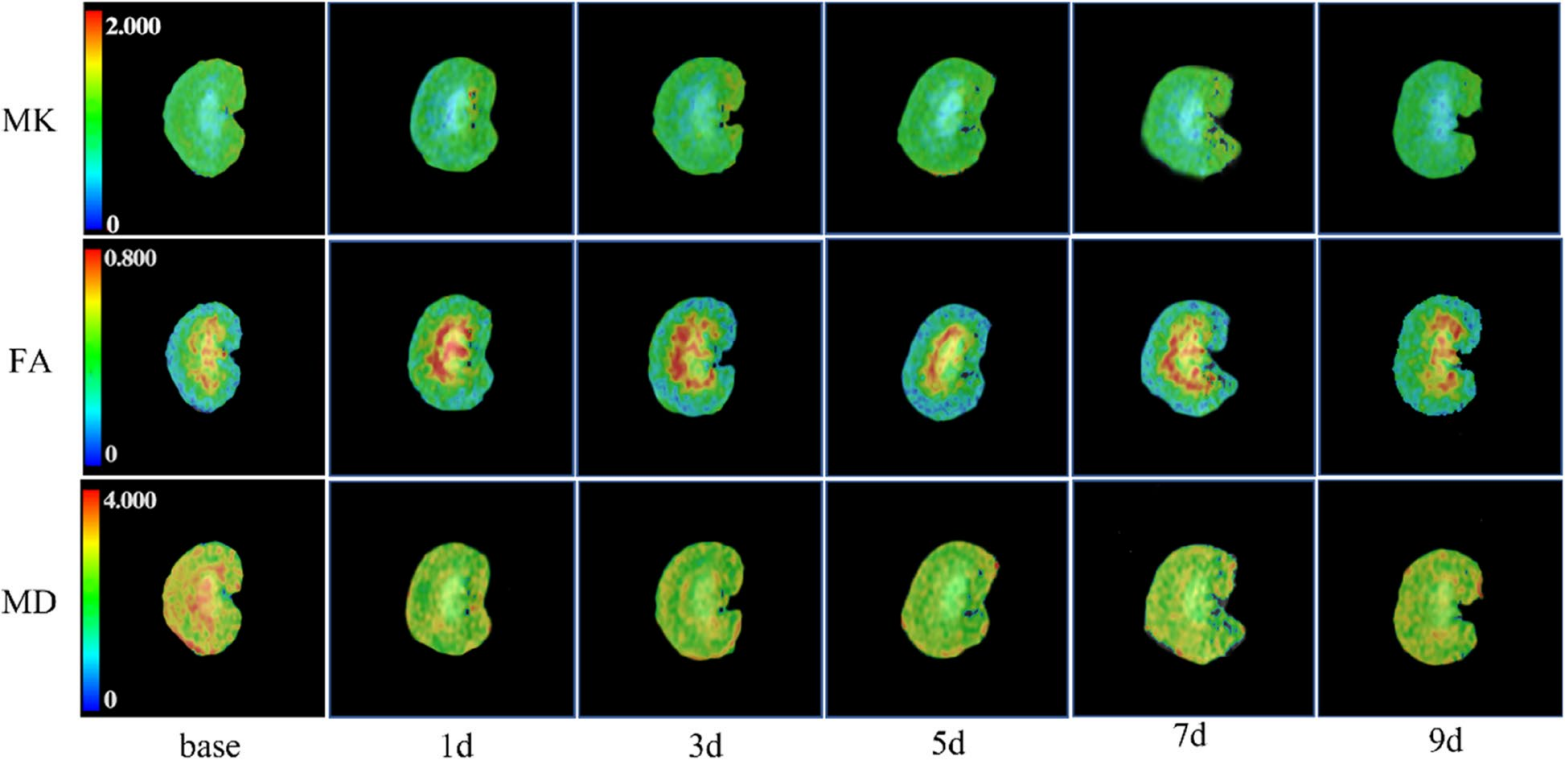
Parametric images of DKI. *MK*, mean kurtosis; *FA*, fractional anisotropy; *MD*, mean diffusivity in different time points; *base*, basement

All ICC values were higher than 0.800. Good agreement was observed between the two radiologists in the HUA group. Thus, the DKI technology was considered to have good agreement in this study (Table 1). Data obtained from the first observer were selected for further statistical analysis. The representative DKI parameters maps at different times are shown in Fig. 2.

**Table 1:** The intraclass correlation coefficient Between two observers of HUA group

		MK	FA	MD
CO	ICC	0.837	0.978	0.974
	95% CI	0.703–0.913	0.958–0.989	0.949–0.986
OS	ICC	0.949	0.948	0.991
	95% CI	0.903–0.974	0.900–0.973	0.982–0.995
IS	ICC	0.990	0.944	0.966
	95% CI	0.980–0.995	0.894–0.971	0.935–0.983

*CO* cortex, *OS* outer stripe of the outer medulla, *IS* inner stripe of the outer medulla, *HUA* hyperuricemia, *ICC* intraclass correlation coefficient, *CI* confidence interval, *MK* mean kurtosis, *MD* mean diffusivity, *FA* fractional anisotropy

**Fig. 3:**
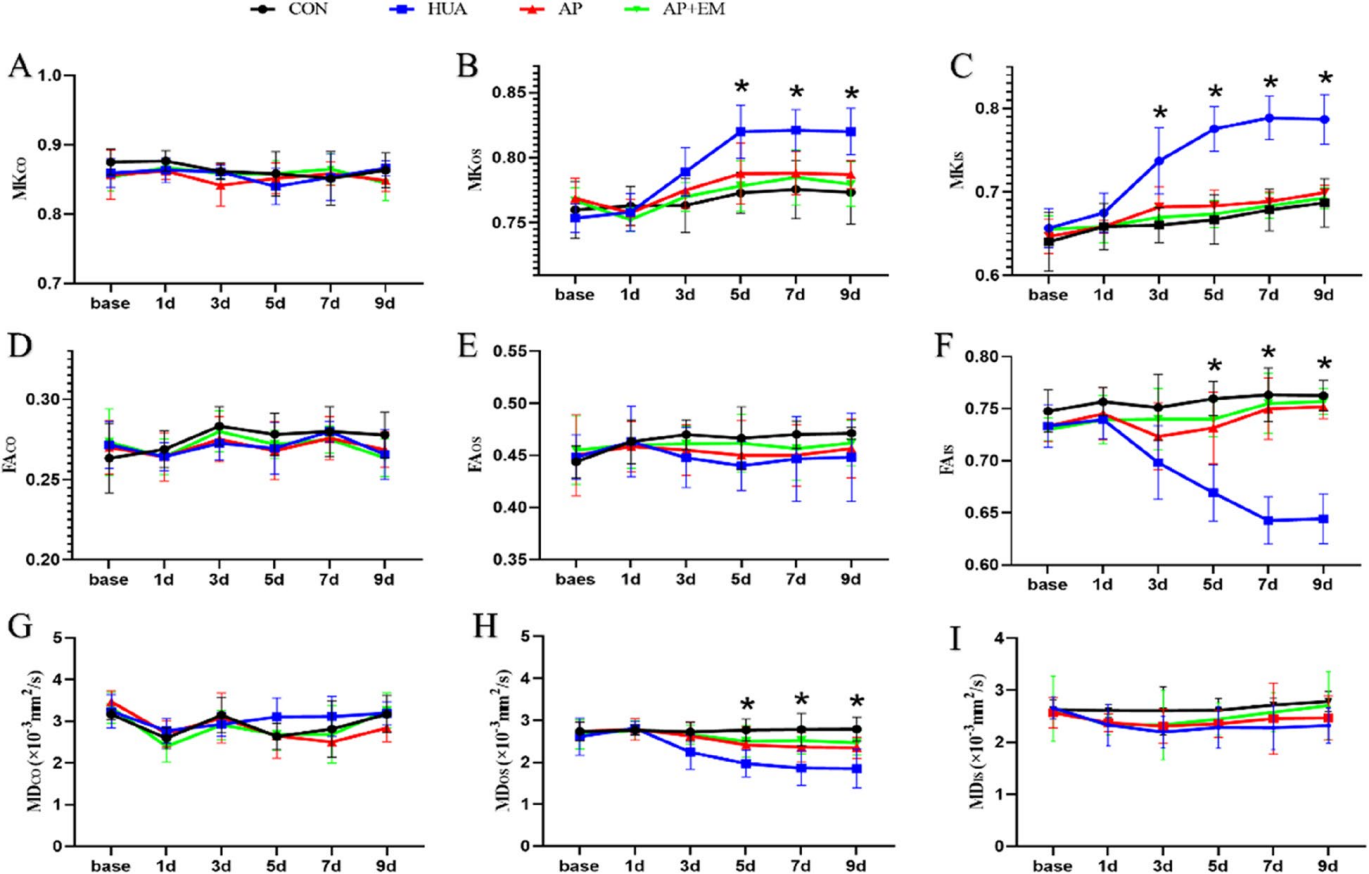
The longitudinal changes of DKI parameters. *, were generated from comparisons between the four groups at each time point by using one-way analysis of variance. *OS*, outer stripe of outer medulla; *IS*, inner stripe of outer medulla; *MK*, mean kurtosis; *FA*, fractional anisotropy; *MD*, mean diffusivity; *base*, basement

In outer medulla, the MK values of HUA, AP, and AP + EM groups gradually increased, with the highest MK values being observed in the HUA group. The MK_OS_ and MK_IS_ values of HUA rats were significantly higher than those of the control rats since day 3 (*P* < 0.05). In OS, the MK values of the AP and AP + EM groups were significantly lower than those of the HUA group from day 5 (*P* < 0.05). In IS, the MK values of AP and AP + EM group were significantly lower than those of HUA group from day 3 (Fig. 3B, C; Table 2; Supplementary Table 1). There was no significant difference in outer medulla between the AP and AP + EM groups (*P* > 0.05). In the cortex, there were no significant differences in MK among the four groups (*P* > 0.05) (Fig. 3A; Table 2; Supplementary Table 1).

**Table 2:** The DKI parameter's values of four groups in different time points (*P*)

**MKCO values**					
	**CON**	**HUA**	**AP**	**AP + EM**	***P***
**base**	0.857 ± 0.018	0.859 ± 0.020	0.857 ± 0.036	0.853 ± 0.020	0.452
**1d**	0.877 ± 0.015	0.864 ± 0.018	0.862 ± 0.012	0.867 ± 0.008	0.291
**3d**	0.862 ± 0.012	0.861 ± 0.010	0.842 ± 0.030	0.859 ± 0.007	0.172
**5d**	0.858 ± 0.032	0.840 ± 0.026	0.852 ± 0.022	0.858 ± 0.019	0.564
**7d**	0.852 ± 0.039	0.853 ± 0.033	0.858 ± 0.017	0.865 ± 0.023	0.859
**9d**	0.863 ± 0.025	0.866 ± 0.011	0.849 ± 0.016	0.845 ± 0.025	0.210
**MKOS values**					
	**CON**	**HUA**	**AP**	**AP + EM**	***P***
**base**	0.760 ± 0.022	0.754 ± 0.011	0.769 ± 0.016	0.767 ± 0.010	0.337
**1d**	0.763 ± 0.015	0.758 ± 0.014	0.758 ± 0.010	0.753 ± 0.009	0.546
**3d**	0.763 ± 0.021	0.789 ± 0.018	0.775 ± 0.014	0.770 ± 0.011	0.074
**5d**	0.773 ± 0.016	0.820 ± 0.020	0.788 ± 0.023	0.778 ± 0.019	0.002*
**7d**	0.776 ± 0.022	0.821 ± 0.016	0.788 ± 0.017	0.785 ± 0.021	0.003*
**9d**	0.773 ± 0.024	0.820 ± 0.018	0.787 ± 0.011	0.780 ± 0.017	0.001*
**MKIS values**					
	**CON**	**HUA**	**AP**	**AP + EM**	***P***
**base**	0.640 ± 0.035	0.657 ± 0.023	0.647 ± 0.021	0.653 ± 0.019	0.679
**1d**	0.658 ± 0.028	0.675 ± 0.023	0.658 ± 0.008	0.658 ± 0.019	0.437
**3d**	0.660 ± 0.021	0.737 ± 0.040	0.682 ± 0.024	0.670 ± 0.013	< 0.001*
**5d**	0.667 ± 0.029	0.776 ± 0.027	0.683 ± 0.019	0.673 ± 0.016	< 0.001*
**7d**	0.678 ± 0.025	0.789 ± 0.026	0.688 ± 0.012	0.681 ± 0.013	< 0.001*
**9d**	0.687 ± 0.029	0.787 ± 0.030	0.699 ± 0.009	0.693 ± 0.0.13	< 0.001*
**FACO values**					
	**CON**	**HUA**	**AP**	**AP + EM**	***P***
**base**	0.263 ± 0.022	0.272 ± 0.015	0.270 ± 0.017	0.273 ± 0.021	0.802
**1d**	0.269 ± 0.011	0.264 ± 0.009	0.264 ± 0.015	0.264 ± 0.011	0.875
**3d**	0.283 ± 0.012	0.273 ± 0.010	0.275 ± 0.014	0.280 ± 0.013	0.462
**5d**	0.278 ± 0.0.13	0.270 ± 0.016	0.268 ± 0.018	0.272 ± 0.009	0.638
**7d**	0.280 ± 0.015	0.280 ± 0.007	0.275 ± 0.013	0.275 ± 0.008	0.810
**9d**	0.278 ± 0.014	0.266 ± 0.016	0.269 ± 0.011	0.264 ± 0.012	0.293
**FAOS values**					
	**CON**	**HUA**	**AP**	**AP + EM**	***P***
**base**	0.444 ± 0.015	0.448 ± 0.021	0.450 ± 0.039	0.455 ± 0.033	0.925
**1d**	0.463 ± 0.021	0.463 ± 0.034	0.459 ± 0.024	0.461 ± 0.022	0.987
**3d**	0.470 ± 0.014	0.448 ± 0.029	0.455 ± 0.024	0.461 ± 0.017	0.371
**5d**	0.467 ± 0.030	0.440 ± 0.024	0.450 ± 0.033	0.462 ± 0.028	0.404
**7d**	0.470 ± 0.013	0.447 ± 0.041	0.450 ± 0.030	0.457 ± 0.031	0.565
**9d**	0.471 ± 0.006	0.448 ± 0.043	0.457 ± 0.028	0.462 ± 0.022	0.553
**FAIS values**					
	**CON**	**HUA**	**AP**	**AP + EM**	***P***
**base**	0.748 ± 0.020	0.733 ± 0.021	0.733 ± 0.015	0.730 ± 0.011	0.316
**1d**	0.757 ± 0.014	0.740 ± 0.018	0.745 ± 0.025	0.739 ± 0.023	0.443
**3d**	0.751 ± 0.032	0.698 ± 0.035	0.723 ± 0.032	0.740 ± 0.030	0.053
**5d**	0.760 ± 0.016	0.669 ± 0.027	0.732 ± 0.034	0.740 ± 0.017	< 0.001*
**7d**	0.763 ± 0.026	0.643 ± 0.023	0.750 ± 0.030	0.755 ± 0.029	< 0.001*
**9d**	0.763 ± 0.014	0.644 ± 0.024	0.752 ± 0.011	0.757 ± 0.012	< 0.001*
**MDCO values (× 10**^**−3**^**mm**^**2**^**/s)**					
	**CON**	**HUA**	**AP**	**AP + EM**	***P***
**base**	3.163 ± 0.118	3.240 ± 0.404	3.468 ± 0.258	3.327 ± 0.371	0.382
**1d**	2.593 ± 0.247	2.772 ± 0.302	2.708 ± 0.309	2.397 ± 0.378	0.206
**3d**	3.147 ± 0.422	2.927 ± 0.289	3.078 ± 0.594	2.910 ± 0.358	0.732
**5d**	2.635 ± 0.315	3.107 ± 0.452	2.652 ± 0.531	2.687 ± 0.377	0.204
**7d**	2.815 ± 0.685	3.115 ± 0.485	2.502 ± 0.097	2.680 ± 0.687	0.285
**9d**	3.170 ± 0.447	3.192 ± 0.281	2.840 ± 0.329	3.262 ± 0.416	0.242
**MDOS values (× 10**^**−3**^**mm**^**2**^**/s)**					
	**CON**	**HUA**	**AP**	**AP + EM**	***P***
**base**	2.735 ± 0.222	2.608 ± 0.447	2.751 ± 0.237	2.666 ± 0.352	0.865
**1d**	2.766 ± 0.121	2.811 ± 0.161	2.784 ± 0.258	2.760 ± 0.235	0.972
**3d**	2.723 ± 0.227	2.239 ± 0.414	2.637 ± 0.328	2.661 ± 0.226	0.052
**5d**	2.770 ± 0.262	1.970 ± 0.318	2.428 ± 0.436	2.503 ± 0.378	0.008*
**7d**	2.781 ± 0.385	1.859 ± 0.413	2.362 ± 0.342	2.517 ± 0.311	0.002*
**9d**	2.790 ± 0.292	1.849 ± 0.460	2.349 ± 0.249	2.463 ± 0.286	0.001*
**MDIS values(× 10**^**−3**^**mm**^**2**^**/s)**					
	**CON**	**HUA**	**AP**	**AP + EM**	***P***
**base**	2.628 ± 0.183	2.660 ± 0.213	2.567 ± 0.296	2.642 ± 0.624	0.975
**1d**	2.607 ± 0.121	2.332 ± 0.401	2.375 ± 0.173	2.333 ± 0.196	0.198
**3d**	2.605 ± 0.463	2.194 ± 0.306	2.307 ± 0.330	2.333 ± 0.658	0.479
**5d**	2.615 ± 0.229	2.287 ± 0.401	2.357 ± 0.266	2.444 ± 0.315	0.316
**7d**	2.716 ± 0.132	2.278 ± 0.421	2.456 ± 0.684	2.580 ± 0.370	0.396
**9d**	2.784 ± 0.194	2.321 ± 0.341	2.468 ± 0.427	2.705 ± 0.650	0.262

Bold characteristics and * were generated from comparisons between the four groups at each time point using one-way ANOVA

*CO* cortex, *OS* outer stripe of the outer medulla, *IS* inner stripe of the outer medulla, *MK* mean kurtosis, *MD* mean diffusivity, *FA* fractional anisotropy, *CON* control, *HUA* hyperuricemia, *AP* allopurinol, *AP* + *EM* allopurinol + empagliflozin, *base* basement

In outer medulla, all groups except the CON group showed a decline trend in FA values, whereas the lowest FA values were shown in HUA group. In IS, the FA values of hyperuricemia rats were significantly lower than those of controls from day 3 after (*P* < 0.05), and the FA_IS_ values of the AP and AP + EM groups were significantly greater than those of the HUA group from day 5 and day 3, respectively (*P* < 0.05) (Fig. 3F; Table 2; Supplementary Table 2). There was no significant difference in the FA_CO_ or FA_OS_ values among the four groups (*P* > 0.05) (Fig. 3D, E; Table 2; Supplementary Table 2).

Unlike the CON group, the MD values declined in the other three groups, with the HUA group having the lowest MD values. From day 3 after the treatment, the MD_OS_ values of the HUA group were significantly lower than those of the control group (*P* < 0.05). The MD_OS_ values of the AP and AP + EM groups were greater than those of the HUA group on day 3 (*P* < 0.05) (Fig. 3H; Table 2; Supplementary Table 3). There were no significant differences in MD_CO_ or MD_IS_ values among the four groups (*P* > 0.05) (Fig. 3G, I; Table 2; Supplementary Table 3).

### Laboratory and Histopathology results

#### SUA, Scr and BUN

The SUA levels of the HUA rats were significantly higher than those of the controls from day 1 (*P* < 0.05). The SUA levels of AP and AP + EM rats were lower after treatment than those of HUA rats, and significantly lower since day 5(*P* < 0.05). There were no significant differences in BUN or Scr levels among four groups (Table 3; Supplementary Table 4).

**Table 3:** Serum levels of uric acid, creatinine, and blood urea nitrogen of four groups at different time points(*P*)

**Uric acid (mg/dL)**					
	**CON**	**HUA**	**AP**	**AP + EM**	***P***
**base**	2.037 ± 0.162	1.960 ± 0.349	2.329 ± 0.353	2.077 ± 0.535	0.664
**1d**	2.000 ± 0.778	4.251 ± 0.585	4.153 ± .0271	4.067 ± 0.181	0.002*
**5d**	1.849 ± 0.407	5.781 ± 0.450	4.868 ± 0.421	4.715 ± 0.434	< 0.001*
**9d**	2.198 ± 0.492	6.042 ± 0.295	5.121 ± 0.368	5.008 ± 0.373	< 0.001*
**Creatinine (μmol/L)**					
	**CON**	**HUA**	**AP**	**AP + EM**	***P***
**base**	25.230 ± 2.561	25.953 ± 2.106	25.277 ± 2.608	28.293 ± 2.507	0.427
**1d**	26.297 ± 2.688	26.303 ± 1.778	27.093 ± 1.306	29.080 ± 1.284	0.286
**5d**	26.790 ± 2.483	25.330 ± 2.455	26.460 ± 4.500	28.207 ± 2.319	0.727
**9d**	28.117 ± 1.636	28.403 ± 3.766	27.330 ± 3.690	28.843 ± 1.917	0.932
**Blood urea nitrogen (mmol/L)**					
	**CON**	**HUA**	**AP**	**AP + EM**	***P***
**base**	4.377 ± 0.374	4.277 ± 0.230	4.770 ± 0.336	4.270 ± 0.336	0.265
**1d**	4.493 ± 0.726	4.173 ± 0.750	4.760 ± 0.344	5.027 ± 0.479	0.398
**5d**	5.083 ± 0.474	4.790 ± 0.322	4.903 ± 0.446	5.530 ± 0.707	0.358
**9d**	4.373 ± 0.211	4.590 ± 0.682	4.673 ± 0.771	5.4033 ± 0.873	0.344

Bold characteristics and *, were generated from comparisons between four groups at each time point by using one-way ANOVA

*CON* control, *HUA* hyperuricemia, *AP* allopurinol, *AP* + *EM* allopurinol + empagliflozin, *base* basement

#### H&E staining

**Fig. 4:**
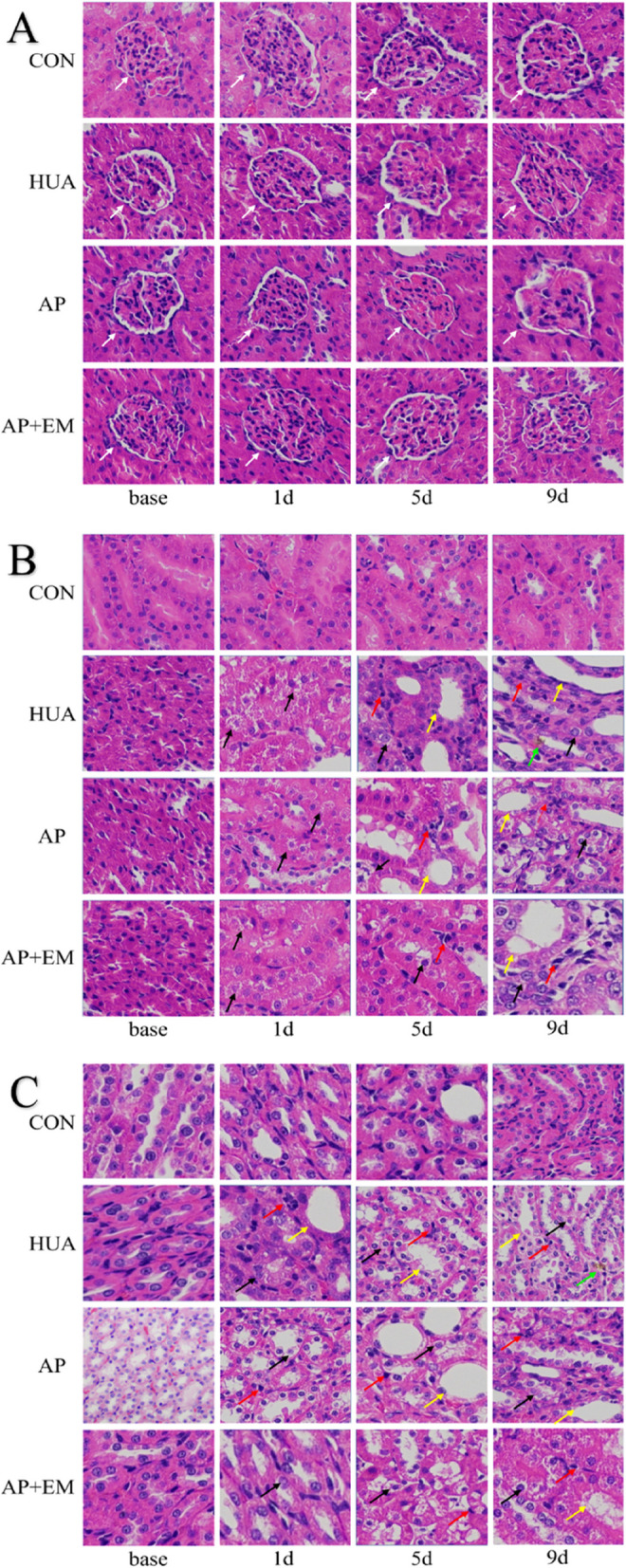
The results of H&E staining. **A **Renal cortex, **B** Renal outer stripe of the renal outer medulla, and **C** Renal inner stripe of the renal outer medulla; 400 × magnification. **A** White arrow, normal renal glomerulus of cortex; **B** and **C**, the swollen tubular epithelial cells (black arrow), dilated renal tubules (yellow arrow), profile of inflammation cells in tubulointerstitial (red arrow), urate crystals in tubulointerstitial (green arrow), and pathological changes gradually aggravated over time; less severe renal tubular injury and declined infiltration of inflammation cells can be observed in the AP and AP + EM groups. *CON*, control; *HUA*, hyperuricemia; *AP*, allopurinol; *AP + EM*, allopurinol + empagliflozin; *base*, basement

In HUA group, swelling tubular epithelial cells, dilated renal tubules, and a profile of inflammatory cells in the tubulointerstitial were observed from day 1, and the abnormality of the renal outer medulla gradually aggravated over time. Furthermore, mildly shrunken renal tubules and deposition of urate crystals in the tubulointerstitial were observed on day 9. Less severe renal tubular injury and decreased infiltration of inflammatory cells were shown in AP and AP + EM groups (Fig. 4B-C). There were no obvious abnormalities in the glomeruli in the renal cortex among the four groups (Fig. 4A).

#### Masson’s trichrome staining

**Fig. 5:**
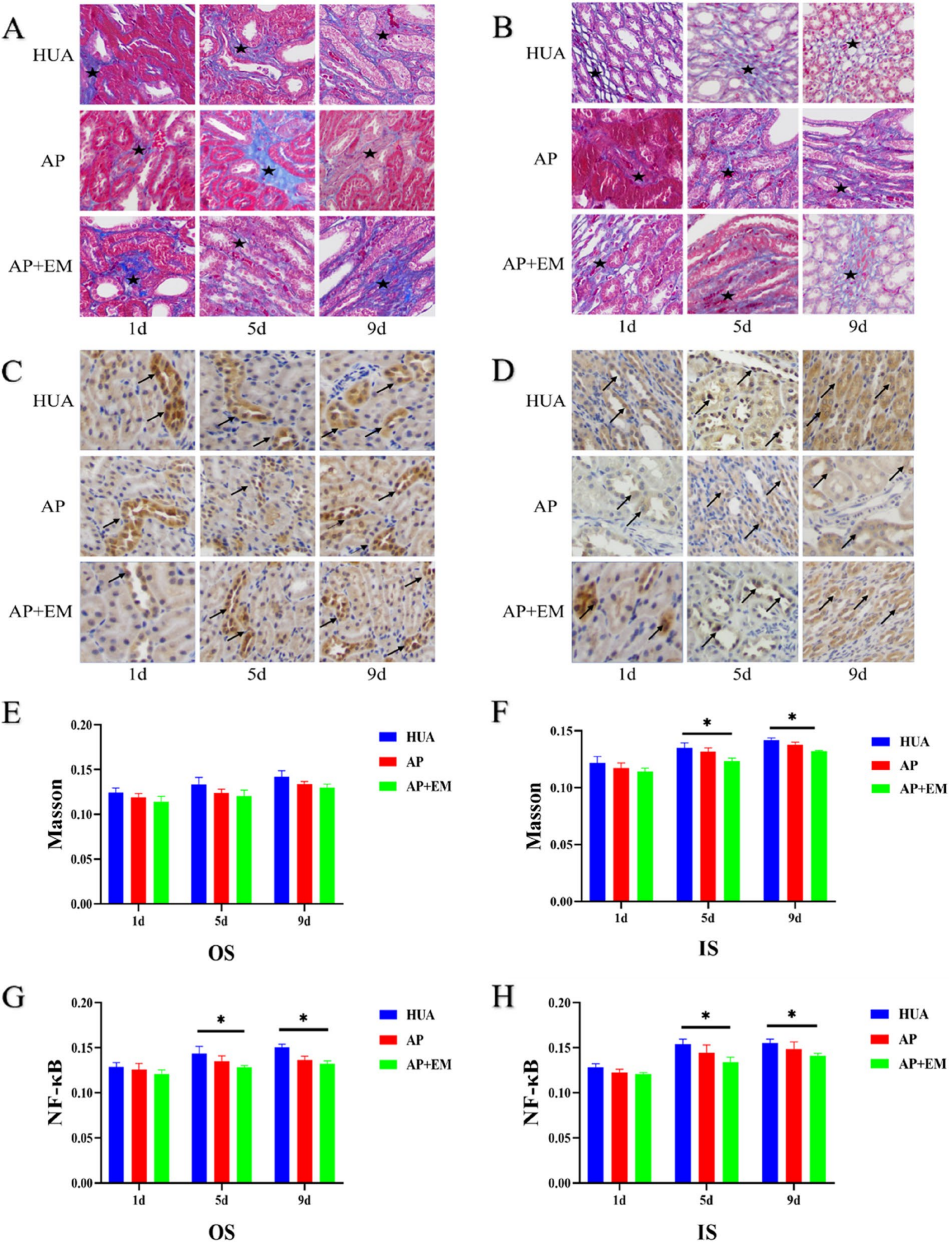
**A** and **B** Renal interstitial fibrosis (black star) on Masson’s trichrome staining of OS and IS (400 × magnification.); **C** and **D** NF-κB expression (black arrow) on immunostaining of OS and IS (400 × magnification.). **E** and **F** The MOD of Masson trichrome staining different group in different time points of OS and IS (400 × magnification.). (G-H) The MOD of NF-κB immunostaining of different group in different time points of OS and IS (400 × magnification.). * generated from comparisons among the four groups at each time point using one-way ANOVA. *OS*, outer stripe of outer medulla; *IS*, inner stripe of outer medulla; *HUA*, hyperuricemia; *AP*, allopurinol; *AP + EM*, allopurinol + empagliflozin; *NF-κB*, nuclear factor kappa B; *MOD,* mean optical density

The results of Masson’s trichrome staining are shown in Fig. 5A-B, E–F. Except for the CON group, the MODs of the other three groups gradually increased in outer medulla, with the highest MOD observed in the HUA group. In OS, the MOD of AP + EM group was significantly lower than that of HUA group since day 1 (*P* < 0.05). In IS, the MOD of the MOD of the AP and AP + EM groups was significantly lower than that of the HUA group from day 5 (*P* < 0.05). (Table 4; Supplementary Table 5).

**Table 4:** The pathological score of four groups in different time points(*P*)

**The MOD of Masson trichrome staining in OS**				
	**HUA**	**AP**	**AP + EM**	***P***
**1d**	0.124 ± 0.005	0.119 ± 0.004	0.114 ± 0.006	0.123
**5d**	0.133 ± 0.008	0.124 ± 0.004	0.121 ± 0.006	0.112
**9d**	0.142 ± 0.007	0.134 ± 0.003	0.130 ± 0.004	0.054
**The MOD of Masson trichrome staining in IS**				
	**HUA**	**AP**	**AP + EM**	***P***
**1d**	0.122 ± 0.006	0.117 ± 0.004	0.114 ± 0.003	0.195
**5d**	0.134 ± 0.004	0.132 ± 0.003	0.123 ± 0.003	0.019*
**9d**	0.142 ± 0.002	0.138 ± 0.002	0.132 ± 0.001	0.002*
**The MOD of NF-κB immunostaining in OS**				
	**HUA**	**AP**	**AP + EM**	***P***
**1d**	0.128 ± 0.005	0.126 ± 0.007	0.121 ± 0.004	0.289
**5d**	0.144 ± 0.008	0.135 ± 0.006	0.128 ± 0.002	0.049*
**9d**	0.151 ± 0.003	0.136 ± 0.004	0.132 ± 0.003	0.002*
**The MOD of NF-κB immunostaining in IS**				
	**HUA**	**AP**	**AP + EM**	***P***
**1d**	0.129 ± 0.004	0.123 ± 0.003	0.121 ± 0.002	0.050
**5d**	0.154 ± 0.006	0.145 ± 0.008	0.134 ± 0.005	0.033*
**9d**	0.155 ± 0.004	0.149 ± 0.008	0.141 ± 0.002	0.047*

Bold characteristics and *, were generated from comparisons among four groups at each time point by using one-way ANOVA

*OS* outer stripe of the outer medulla, *IS* inner stripe of the outer medulla, *CON* control, *HUA* hyperuricemia, *AP* allopurinol, *AP* + *EM* allopurinol + empagliflozin, *MOD* mean optical density

#### NF-κB immunostaining

The results of NF-κB immunostaining are shown in Fig. 5C-D, G-H. In outer medulla, the MOD in the HUA, AP, and AP + EM groups gradually increased, and the HUA group showed the highest result. In OS, the MOD of AP + EM group was significantly lower than that of HUA group since day 5 and the MOD of AP group was significantly lower than that of HUA group on day 9 (*P* < 0.05). In IS, the MOD of the AP + EM group was significantly lower than that of the HUA group since day 1 (*P* < 0.05) (Table 4; Supplementary Table 6).

#### Correlation analysis

**Fig. 6:**
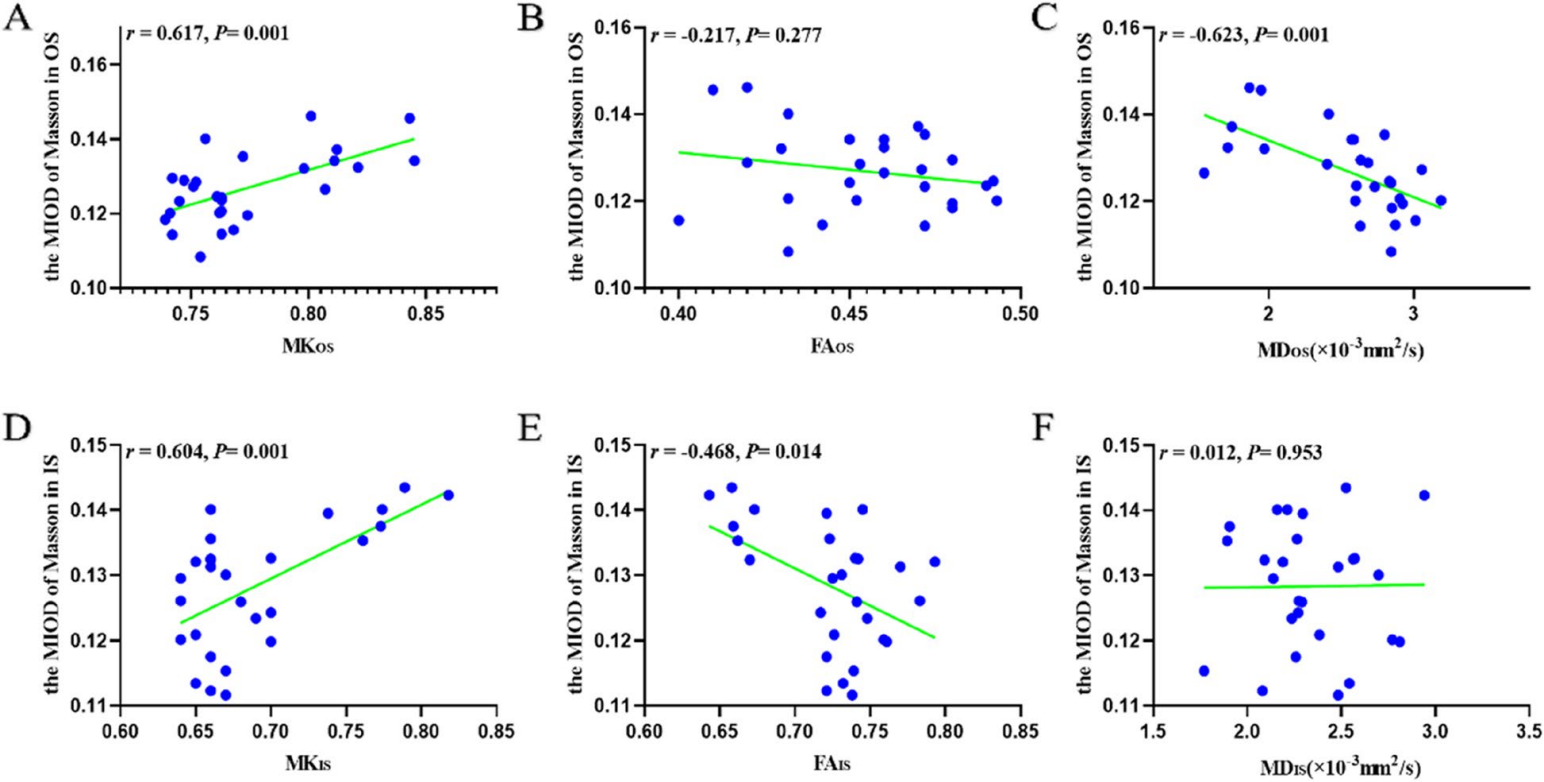
Correlational analyses of the MOD of Masson trichrome staining with DKI parameters. *OS*, outer stripe of outer medulla; *IS*, inner stripe of outer medulla; *MK*, mean kurtosis; *FA*, fractional anisotropy; *MD*, mean diffusivity; *MOD*, mean optical density

A positive correlation between the MK values and Masson’s trichrome staining results was observed (*r* = 0.687, *P* < 0.001; *r* = 0.604,* P* = 0.001) in outer medulla. The MD_OS_ and FA_IS_ values negatively correlated with the MOD of Masson’s trichrome staining (*r* = -0.626,* P* < 0.001; *r* = -0.468,* P* = 0.014, respectively) (Fig. 6).

## Discussion

In this study, not only the DKI was used to assess hyperuricemia-induced early renal fibrosis, but the alterations in renal fibrosis after urate-lowering therapy (ULT) were also monitor as well. Our results showed that hyperuricemia-induced renal injury, which mainly occurred in the renal medulla, and renal fibrosis became increasingly serious over time. The damage to renal tissue can be evaluated by using quantitative parameters of DKI, specifically the MK value. Additionally, positive correlations were detected between the MOD of Masson’s trichrome staining and MK_OS_ and MK_IS_ values. Therefore, we propose that DKI is a non-invasive method for monitoring the progression of renal fibrosis caused by hyperuricemia.

In terms of the b-value setting for abdominal DKI sequences, Pentang et al. found that deviations from a mono-exponential decay were observed in the diffusion signal when b-values were in the range of 600–800 s/mm^2^ [27]. This suggests that 800 s/mm^2^ is sufficient to capture non-Gaussian diffusion. Given that higher b-values can diminish the SNR of diffusion images, we select 800 s/mm^2^ as the high b-value in the DKI acquisition. Moreover, the intravoxel incoherent motion (IVIM) effect can be visible at very low b-values (typically below 400 s/mm^2^), potentially contaminating DKI measurements. To address this, prior studies recommend employing b-values ≥ 200 s/mm^2^in DKI acquisition, which helps mitigate the influence of capillary perfusion [28, 29]. Hence, a b-value of 400 s/mm^2^ was chosen as the minimal b-value.

In this study, the MK_OS,_ MK_IS_, FA_IS,_ and MD_OS_ between the HUA and control groups were significantly different from those on day 3, probably because of the different renal tissue microstructures induced by hyperuricemia. Based on the results of this study, the infiltration of inflammatory cells into the renal interstitial, progressive tubular cell atrophy, and tubular dilation were observed, which is consistent with previous studies [18, 30]. This can lead to more complex microstructures and restrict the diffusion of water molecules. Hence, the MK value increased, and the MD value decreased. Furthermore, according to Mao et al. [22] and Li et al. [18], aggravated renal interstitial fibrosis could be another reason for the higher MK values and lower MD, and the FA values reflect the movement of water molecules, which is related to the renal tubular arrangement [19, 22]. In this study, renal tubular damage such as tubular epithelial cell injury and expanded renal tubules was observed in the outer medulla of the HUA group, which may have led to a decreased FA value. According to some studies, renal interstitial fibrosis might be another important reason for the lower FA values, which is similar to the results of this study [18, 22].

Compared with the HUA group, the AP and AP + EM groups had lower MK values and higher FA and MD values in the outer medulla. This was probably due to the therapeutic effects of AP and AP + EM. First, it can be attributed to the ULT. High SUA levels will cause renal injury [5, 6], while ULT can alleviate the UA-induced renal damage [14, 15, 31–33]. Additionally, allopurinol inhibits NLRP3/NF-κB inflammasome activation and reduces renal fibrosis [12–15]. Hence, in comparison to the HUA group, the AP and AP + EM groups showed decreased NF-κB expression, less infiltration of inflammatory cells, and reduced renal fibrosis. A lower SUA level leads to relatively mild renal tubule injury, which is consistent with the findings of other studies [34, 35]. The degree of kidney injury was lower in the AP and AP + EM groups than in the HUA group, which led to lower MK values and higher MD and FA values in the AP and AP + EM groups. Only a slight difference was observed between the AP and AP + EM groups, which is probably because the combination reduces the SUA effect of AP and SGLT-2i and causes a more prominent renal protective effect.

Some studies indicated that the DKI showed a great potnetional to monitor the renal fibrosis in humans such as CKD [22], IgA nephropathy [19], or the kidney with impaired renal function [30]. The performance of animal studies also suggests that DKI is a valuable and potential method for noninvasive renal fibrosis assessment [18, 36]. These suggested that DKI is a feasible technique for evaluating renal fibrosis. In this study, DKI was used to assess renal interstitial fibrosis in hyperuricemia-induced renal injury. Positive correlations were found between the MK values and the MOD of Masson’s trichrome staining. Contrastingly, negative correlations were found between the MD_OS_ and FA_IS_ values and the MOD of Masson’s trichrome staining. This is consistent with other studies [18, 19, 22]. Thus, DKI can be used as a potential tool to evaluate renal interstitial fibrosis in hyperuricemia-induced early kidney injury. Furthermore, the correlation between the MK_OS,_ MK_IS_ values, and the MOD of Masson’s trichrome staining (*r* = 0.687, *P* < 0.001; *r* = 0.604,* P* = 0.001) was stronger than that between the FA_IS_ and MD_OS_ values and the MOD of Masson’s trichrome staining (*r* = -0.468,* P* = 0.014;* r* = -0.626,* P* < 0.001). This indicates that MK is more sensitive than FA and MD in detecting renal interstitial fibrosis, which is consistent with the findings of Liu et al. [19].

Currently, Scr or BUN is the most commonly used method to monitor the glomerular filtration rate and reflect renal function. In this study, no significant difference was observed between the Scr or BUN of our four groups, but some pathological changes and alterations in DKI parametric values were observed, which indicates that DKI is a more sensitive technique for detecting renal injury.

### Limitations

The limitations of this study are as follows. First, the ROIs cannot perfectly match with the location of histological analysis in current study, which might slightly influence analysis results. In the further, we would adopt a preferable method which has been employed by Kjølby et al. [36] to acquire a more accurate result. Second, SGLT-2i treatment was not evaluated independently due to the lack of a single SGLT-2i treatment group. We will set up a future study including a single SGLT-2i treatment group for further comparison of the therapeutic effects. Third, the sample size was relatively small. Further studies with larger sample sizes are required to confirm these findings.

## Conclusions

In conclusion, DKI parameters are correlated with the renal fibrosis index of Masson’s trichrome staining demonstrated that DKI might serve as a feasible and sensitive technique to assess hyperuricemia-induced early renal fibrosis. This may provide an accurate and promising MRI technique for assessing hyperuricemia-induced renal fibrosis and alterations after therapy, which will be helpful for clinical treatment and follow-up.

### Supplementary Information

**Supplementary Material 1.**

## Data Availability

The datasets used and/or analyzed during the current study are available from the corresponding author on reasonable request.

## Abbreviations

DKI: Diffusion kurtosis imaging
CON: Control
HUA: Hyperuricemia
AP: Allopurinol
EM: Empagliflozin
NF-κB: Nuclear factor kappa B
H&E: Hematoxylin and eosin
CO: Cortex
OS: Outer stripe of outer medulla
IS: Inner stripe of outer medulla
MK: Mean kurtosis
FA: Fractional anisotropy
MD: Mean diffusivity
SUA: Serum uric acid
BUN: Blood urea nitrogen
Scr: Serum creatinine
MOD: Mean optical density
ULT: Urate-lowering therapy

## References

[1] Liu N, Xu H, Sun Q, Yu X, Chen W, Wei H, Jiang J, Xu Y, Lu W (2021). The role of oxidative stress in hyperuricemia and Xanthine Oxidoreductase (XOR) inhibitors. Oxid Med Cell Longev.

[2] Wang Z, Li Y, Liao W, Huang J, Liu Y, Li Z, Tang J (2022). Gut microbiota remodeling: a promising therapeutic strategy to confront hyperuricemia and gout. Front Cell Infect Microbiol.

[3] Dehlin M, Jacobsson L, Roddy E (2020). Global epidemiology of gout: prevalence, incidence, treatment patterns and risk factors. Nat Rev Rheumatol.

[4] Ejaz AA, Nakagawa T, Kanbay M, Kuwabara M, Kumar A, Garcia Arroyo FE, Roncal-Jimenez C, Sasai F, Kang DH, Jensen T (2020). Hyperuricemia in kidney disease: a major risk factor for cardiovascular events, vascular calcification, and renal damage. Semin Nephrol.

[5] Zhou X, Zhang B, Zhao X, Lin Y, Wang J, Wang X, Hu N, Wang S (2021). Chlorogenic acid supplementation ameliorates hyperuricemia, relieves renal inflammation, and modulates intestinal homeostasis. Food Funct.

[6] Gherghina ME, Peride I, Tiglis M, Neagu TP, Niculae A, Checherita IA (2022). Uric Acid and Oxidative Stress-Relationship with Cardiovascular, Metabolic, and Renal Impairment. Int J Mol Sci..

[7] Pan Z, Yang K, Wang H, Xiao Y, Zhang M, Yu X, Xu T, Bai T, Zhu H (2020). MFAP4 deficiency alleviates renal fibrosis through inhibition of NF-κB and TGF-β/Smad signaling pathways. FASEB J.

[8] Xiao H, Sun X, Liu R, Chen Z, Lin Z, Yang Y, Zhang M, Liu P, Quan S, Huang H (2020). Gentiopicroside activates the bile acid receptor Gpbar1 (TGR5) to repress NF-kappaB pathway and ameliorate diabetic nephropathy. Pharmacol Res.

[9] Park JH, Jo YI, Lee JH (2020). Renal effects of uric acid: hyperuricemia and hypouricemia. Korean J Intern Med.

[10] Dalbeth N, Gosling AL, Gaffo A, Abhishek A (2021). Gout. Lancet.

[11] Danve A, Sehra ST, Neogi T (2021). Role of diet in hyperuricemia and gout. Best Pract Res Clin Rheumatol.

[12] Chen L, Lan Z (2017). Polydatin attenuates potassium oxonate-induced hyperuricemia and kidney inflammation by inhibiting NF-κB/NLRP3 inflammasome activation via the AMPK/SIRT1 pathway. Food Funct.

[13] Liu ZQ, Sun X, Liu ZB, Zhang T, Zhang LL, Wu CJ (2022). Phytochemicals in traditional Chinese medicine can treat gout by regulating intestinal flora through inactivating NLRP3 and inhibiting XOD activity. J Pharm Pharmacol.

[14] Foresto-Neto O, Ávila VF, Arias SCA, Zambom FFF, Rempel LCT, Faustino VD, Machado FG, Malheiros D, Abensur H, Camara NOS (2018). NLRP3 inflammasome inhibition ameliorates tubulointerstitial injury in the remnant kidney model. Lab Invest..

[15] Haryono A, Nugrahaningsih DAA, Sari DCR, Romi MM, Arfian N (2018). Reduction of serum uric acid associated with attenuation of renal injury, inflammation and macrophages M1/M2 ratio in hyperuricemic mice model. Kobe J Med Sci.

[16] Zhao Y, Xu L, Tian D, Xia P, Zheng H, Wang L, Chen L (2018). Effects of sodium-glucose co-transporter 2 (SGLT2) inhibitors on serum uric acid level: a meta-analysis of randomized controlled trials. Diabetes Obes Metab.

[17] Suijk DLS, van Baar MJB, van Bommel EJM, Iqbal Z, Krebber MM, Vallon V, Touw D, Hoorn EJ, Nieuwdorp M, Kramer MMH (2022). SGLT2 inhibition and uric acid excretion in patients with type 2 diabetes and normal kidney function. Clin J Am Soc Nephrol.

[18] Li A, Liang L, Liang P, Hu Y, Xu C, Hu X, Shen Y, Hu D, Li Z, Kamel IR (2020). Assessment of renal fibrosis in a rat model of unilateral ureteral obstruction with diffusion kurtosis imaging: comparison with α-SMA expression and (18)F-FDG PET. Magn Reson Imaging.

[19] Liu Y, Zhang GM, Peng X, Wen Y, Ye W, Zheng K, Li X, Sun H, Chen L (2018). Diffusional kurtosis imaging in assessing renal function and pathology of IgA nephropathy: a preliminary clinical study. Clin Radiol.

[20] Bonani M, Seeger H, Weber N, Lorenzen JM, Wüthrich RP, Kistler AD (2021). Safety of kidney biopsy when performed as an outpatient procedure. Kidney Blood Press Res.

[21] Zhou H, Zhang J, Zhang XM, Chen T, Hu J, Jing Z, Jian S (2021). Noninvasive evaluation of early diabetic nephropathy using diffusion kurtosis imaging: an experimental study. Eur Radiol.

[22] Mao W, Ding Y, Ding X, Fu C, Zeng M, Zhou J (2021). Diffusion kurtosis imaging for the assessment of renal fibrosis of chronic kidney disease: a preliminary study. Magn Reson Imaging.

[23] Johnson RJ, Bakris GL, Borghi C, Chonchol MB, Feldman D, Lanaspa MA, Merriman TR, Moe OW, Mount DB, Sanchez Lozada LG (2018). Hyperuricemia, acute and chronic kidney disease, hypertension, and cardiovascular disease: report of a scientific workshop organized by the National Kidney Foundation. Am J Kidney Dis.

[24] Jensen JH, Helpern JA, Ramani A, Lu H, Kaczynski K (2005). Diffusional kurtosis imaging: the quantification of non-gaussian water diffusion by means of magnetic resonance imaging. Magn Reson Med.

[25] Hu G, Liang W, Wu M, Lai C, Mei Y, Li Y, Xu J, Luo L, Quan X (2019). Comparison of T1 Mapping and T1rho values with conventional diffusion-weighted imaging to assess fibrosis in a rat model of unilateral ureteral obstruction. Acad Radiol.

[26] Wang B, Wang Y, Li L, Guo J, Wu PY, Zhang H, Zhang H (2022). Diffusion kurtosis imaging and arterial spin labeling for the noninvasive evaluation of persistent post-contrast acute kidney injury. Magn Reson Imaging.

[27] Pentang G, Lanzman RS, Heusch P, Müller-Lutz A, Blondin D, Antoch G, Wittsack HJ (2014). Diffusion kurtosis imaging of the human kidney: a feasibility study. Magn Reson Imaging.

[28] Rosenkrantz AB, Padhani AR, Chenevert TL, Koh DM, De Keyzer F, Taouli B, Le Bihan D (2015). Body diffusion kurtosis imaging: Basic principles, applications, and considerations for clinical practice. J Magn Reson Imaging.

[29] Filli L, Wurnig M, Nanz D, Luechinger R, Kenkel D, Boss A (2014). Whole-body diffusion kurtosis imaging: initial experience on non-Gaussian diffusion in various organs. Invest Radiol.

[30] Li A, Yuan G, Hu Y, Shen Y, Hu X, Hu D, Li Z (2022). Renal functional and interstitial fibrotic assessment with non-Gaussian diffusion kurtosis imaging. Insights Imaging.

[31] La Grotta R, de Candia P, Olivieri F, Matacchione G, Giuliani A, Rippo MR, Tagliabue E, Mancino M, Rispoli F, Ferroni S (2022). Anti-inflammatory effect of SGLT-2 inhibitors via uric acid and insulin. Cell Mol Life Sci.

[32] Abdollahi E, Keyhanfar F, Delbandi AA, Falak R, Hajimiresmaiel SJ, Shafiei M (2022). Dapagliflozin exerts anti-inflammatory effects via inhibition of LPS-induced TLR-4 overexpression and NF-κB activation in human endothelial cells and differentiated macrophages. Eur J Pharmacol.

[33] Bailey CJ, Day C, Bellary S (2022). Renal protection with SGLT2 inhibitors: effects in acute and chronic kidney disease. Curr DiabRep.

[34] He L, Fan Y, Xiao W, Chen T, Wen J, Dong Y, Wang Y, Li S, Xue R, Zheng L (2017). Febuxostat attenuates ER stress mediated kidney injury in a rat model of hyperuricemic nephropathy. Oncotarget.

[35] Wang Y, Kong W, Wang L, Zhang T, Huang B, Meng J, Yang B, Xie Z, Zhou H (2020). Multiple-purpose connectivity map analysis reveals the benefits of Esculetin to hyperuricemia and renal fibrosis. Int J Mol Sci.

[36] Kjølby BF, Khan AR, Chuhutin A, Pedersen L, Jensen JB, Jakobsen S, Zeidler D, Sangill R, Nyengaard JR, Jespersen SN (2016). Fast diffusion kurtosis imaging of fibrotic mouse kidneys. NMR Biomed.

