# Supplementary material for: Renal interstitial fibrotic assessment using non-Gaussian diffusion kurtosis imaging in a rat model of hyperuricemia
Source: BMC Med Imaging. 2024 Apr 3;24:78. doi: 10.1186/s12880-024-01259-8 (PMC10988851; doi:10.1186/s12880-024-01259-8)
Supplement: Supplementary file 1 — Supplementary Material 1. [file 12880_2024_1259_MOESM1_ESM.docx]

| **Supplementary table 1 LSD as a post hoc test of one-way ANOVA for the MK value of the four groups at different time points (*P*)** | | | | | | | | | | | | | | |
| --- | --- | --- | --- | --- | --- | --- | --- | --- | --- | --- | --- | --- | --- | --- |
|  | **MKCO** | | | |  | **MKOS** | | | |  | **MKIS** | | | |
|  | **CON** | **HUA** | **AP** | **AP+EM** |  | **CON** | **HUA** | **AP** | **AP+EM** |  | **CON** | **HUA** | **AP** | **AP+EM** |
| **base** |  |  |  |  |  |  |  |  |  |  |  |  |  |  |
| **CON** | - |  |  |  |  | - |  |  |  |  | - |  |  |  |
| **HUA** | 0.283 | - |  |  |  | 0.484 | - |  |  |  | 0.267 | - |  |  |
| **AP** | 0.211 | 0.853 | - |  |  | 0.340 | 0.106 | - |  |  | 0.653 | 0.501 | - |  |
| **AP+EM** | 0.143 | 0.677 | 0.817 | - |  | 0.450 | 0.153 | 0.838 | - |  | 0.372 | 0.822 | 0.653 | - |
| **1d** |  |  |  |  |  |  |  |  |  |  |  |  |  |  |
| **CON** | - |  |  |  |  | - |  |  |  |  | - |  |  |  |
| **HUA** | 0.113 | - |  |  |  | 0.504 | - |  |  |  | 0.184 | - |  |  |
| **AP** | 0.087 | 0.885 | - |  |  | 0.475 | 0.963 | - |  |  | 1.000 | 0.184 | - |  |
| **AP+EM** | 0.260 | 0.625 | 0.527 | - |  | 0.155 | 0.434 | 0.461 | - |  | 1.000 | 0.184 | 1.000 | - |
| **3d** |  |  |  |  |  |  |  |  |  |  |  |  |  |  |
| **CON** | - |  |  |  |  | - |  |  |  |  | - |  |  |  |
| **HUA** | 0.946 | - |  |  |  | **0.013*** | - |  |  |  | **<0.001*** | - |  |  |
| **AP** | 0.057 | 0.065 | - |  |  | 0.232 | 0.150 | - |  |  | 0.167 | **0.002** | - |  |
| **AP+EM** | 0.783 | 0.836 | 0.097 | - |  | 0.489 | 0.056 | 0.603 | - |  | 0.539 | **<0.001*** | 0.427 | - |
| **5d** |  |  |  |  |  |  |  |  |  |  |  |  |  |  |
| **CON** | - |  |  |  |  | - |  |  |  |  | - |  |  |  |
| **HUA** | 0.225 | - |  |  |  | **0.001*** | - |  |  |  | **<0.001*** | - |  |  |
| **AP** | 0.654 | 0.435 | - |  |  | 0.210 | **0.011*** | - |  |  | 0.233 | **<0.001*** | - |  |
| **AP+EM** | 1.000 | 0.225 | 0.654 | - |  | 0.646 | **0.002*** | 0.417 | - |  | 0.628 | **<0.001*** | 0.469 | - |
| **7d** |  |  |  |  |  |  |  |  |  |  |  |  |  |  |
| **CON** | - |  |  |  |  | - |  |  |  |  | - |  |  |  |
| **HUA** | 0.922 | - |  |  |  | **0.001*** | - |  |  |  | **<0.001*** | - |  |  |
| **AP** | 0.697 | 0.770 | - |  |  | 0.273 | **0.007** | - |  |  | 0.399 | **<0.001*** | - |  |
| **AP+EM** | 0.438 | 0.497 | 0.697 | - |  | 0.410 | **0.004** | 0.778 | - |  | 0.777 | **<0.001*** | 0.572 | - |
| **9d** |  |  |  |  |  |  |  |  |  |  |  |  |  |  |
| **CON** | - |  |  |  |  | - |  |  |  |  | - |  |  |  |
| **HUA** | 0.802 | - |  |  |  | **<0.001*** | - |  |  |  | **<0.001*** | - |  |  |
| **AP** | 0.219 | 0.144 | - |  |  | 0.202 | **0.005** | - |  |  | 0.344 | **<0.001*** | - |  |
| **AP+EM** | 0.132 | 0.083 | 0.765 | - |  | 0.553 | **0.001*** | 0.483 | - |  | 0.643 | **<0.001*** | 0.624 | - |

Note: Bold characteristic and *, significant difference between groups. CO, cortex; OS, outer stripe of the outer medulla; IS, inner stripe of the outer medulla; MK, mean kurtosis; MD, mean diffusivity; FA, fractional anisotropy; CON, control; HUA, hyperuricemia; AP, allopurinol; AP+EM, allopurinol+empagliflozin; base, basement.

| **Supplementary table 2 LSD as a post hoc test of one-way ANOVA for the FA value of the four groups at different time points (*P)*** | | | | | | | | | | | | | | |
| --- | --- | --- | --- | --- | --- | --- | --- | --- | --- | --- | --- | --- | --- | --- |
|  | **FACO** | | | |  | **FAOS** | | | |  | **FAIS** | | | |
|  | **CON** | **HUA** | **AP** | **AP+EM** |  | **CON** | **HUA** | **AP** | **AP+EM** |  | **CON** | **HUA** | **AP** | **AP+EM** |
| **base** |  |  |  |  |  |  |  |  |  |  |  |  |  |  |
| **CON** | - |  |  |  |  | - |  |  |  |  | - |  |  |  |
| **HUA** | 0.448 | - |  |  |  | 0.788 | - |  |  |  | 0.165 | - |  |  |
| **AP** | 0.543 | 0.878 | - |  |  | 0.713 | 0.921 | - |  |  | 0.165 | 1.000 | - |  |
| **AP+EM** | 0.364 | 0.878 | 0.760 | - |  | 0.507 | 0.691 | 0.766 | - |  | 0.091 | 0.741 | 0.741 | - |
| **1d** |  |  |  |  |  |  |  |  |  |  |  |  |  |  |
| **CON** | - |  |  |  |  | - |  |  |  |  | - |  |  |  |
| **HUA** | 0.517 | - |  |  |  | 0.991 | - |  |  |  | 0.167 | - |  |  |
| **AP** | 0.502 | 0.981 | - |  |  | 0.766 | 0.758 | - |  |  | 0.343 | 0.648 | - |  |
| **AP+EM** | 0.502 | 0.981 | 1.000 | - |  | 0.860 | 0.851 | 0.903 | - |  | 0.159 | 0.978 | 0.628 | - |
| **3d** |  |  |  |  |  |  |  |  |  |  |  |  |  |  |
| **CON** | - |  |  |  |  | - |  |  |  |  | - |  |  |  |
| **HUA** | 0.152 | - |  |  |  | 0.095 | - |  |  |  | **0.010*** | - |  |  |
| **AP** | 0.268 | 0.731 | - |  |  | 0.250 | 0.577 | - |  |  | 0.151 | 0.195 | - |  |
| **AP+EM** | 0.647 | 0.318 | 0.507 | - |  | 0.485 | 0.310 | 0.640 | - |  | 0.556 | **0.037*** | 0.382 | - |
| **5d** |  |  |  |  |  |  |  |  |  |  |  |  |  |  |
| **CON** | - |  |  |  |  | - |  |  |  |  | - |  |  |  |
| **HUA** | 0.312 | - |  |  |  | 0.127 | - |  |  |  | **<0.001*** | - |  |  |
| **AP** | 0.238 | 0.859 | - |  |  | 0.331 | 0.557 | - |  |  | 0.065 | **<0.001*** | - |  |
| **AP+EM** | 0.469 | 0.768 | 0.638 | - |  | 0.768 | 0.210 | 0.494 | - |  | 0.185 | **<0.001*** | 0.567 | - |
| **7d** |  |  |  |  |  |  |  |  |  |  |  |  |  |  |
| **CON** | - |  |  |  |  | - |  |  |  |  | - |  |  |  |
| **HUA** | 1.000 | - |  |  |  | 0.197 | - |  |  |  | **<0.001*** | - |  |  |
| **AP** | 0.538 | 0.538 | - |  |  | 0.266 | 0.851 | - |  |  | 0.400 | **<0.001*** | - |  |
| **AP+EM** | 0.461 | 0.461 | 0.902 | - |  | 0.454 | 0.574 | 0.707 | - |  | 0.597 | **<0.001*** | 0.750 | - |
| **9d** |  |  |  |  |  |  |  |  |  |  |  |  |  |  |
| **CON** | - |  |  |  |  | - |  |  |  |  | - |  |  |  |
| **HUA** | 0.129 | - |  |  |  | 0.168 | - |  |  |  | **<0.001*** | - |  |  |
| **AP** | 0.247 | 0.700 | - |  |  | 0.373 | 0.610 | - |  |  | 0.269 | **<0.001*** | - |  |
| **AP+EM** | 0.080 | 0.797 | 0.522 | - |  | 0.568 | 0.406 | 0.744 | - |  | 0.552 | **<0.001*** | 0.600 | - |

Note: Bold characteristic and *, significant difference between groups. CO, cortex; OS, outer stripe of the outer medulla; IS, inner stripe of the outer medulla; MK, mean kurtosis; MD, mean diffusivity; FA, fractional anisotropy; CON, control; HUA, hyperuricemia; AP, allopurinol; AP+EM, allopurinol+empagliflozin; base, basement.

| **Supplementary table 3 LSD as a post hoc test of one-way ANOVA for the MD value of the four groups at different time points P** | | | | | | | | | | | | | | |
| --- | --- | --- | --- | --- | --- | --- | --- | --- | --- | --- | --- | --- | --- | --- |
|  | **MDCO** | | | |  | **MDOS** | | | |  | **MDIS** | | | |
|  | **CON** | **HUA** | **AP** | **AP+EM** |  | **CON** | **HUA** | **AP** | **AP+EM** |  | **CON** | **HUA** | **AP** | **AP+EM** |
| **base** |  |  |  |  |  |  |  |  |  |  |  |  |  |  |
| **CON** | - |  |  |  |  | - |  |  |  |  | - |  |  |  |
| **HUA** | 0.672 | - |  |  |  | 0.511 | - |  |  |  | 0.884 | - |  |  |
| **AP** | 0.103 | 0.215 | - |  |  | 0.933 | 0.459 | - |  |  | 0.779 | 0.671 | - |  |
| **AP+EM** | 0.371 | 0.632 | 0.436 | - |  | 0.718 | 0.764 | 0.657 | - |  | 0.951 | 0.933 | 0.733 | - |
| **1d** |  |  |  |  |  |  |  |  |  |  |  |  |  |  |
| **CON** | - |  |  |  |  | - |  |  |  |  | - |  |  |  |
| **HUA** | 0.335 | - |  |  |  | 0.703 | - |  |  |  | 0.068 | - |  |  |
| **AP** | 0.531 | 0.729 | - |  |  | 0.877 | 0.820 | - |  |  | 0.120 | 0.765 | - |  |
| **AP+EM** | 0.289 | 0.051 | 1.000 | - |  | 0.961 | 0.667 | 0.839 | - |  | 0.069 | 0.995 | 0.770 | - |
| **3d** |  |  |  |  |  |  |  |  |  |  |  |  |  |  |
| **CON** | - |  |  |  |  | - |  |  |  |  | - |  |  |  |
| **HUA** | 0.387 | - |  |  |  | **0.013*** | - |  |  |  | 0.138 | - |  |  |
| **AP** | 0.786 | 0.549 | - |  |  | 0.632 | **0.038*** | - |  |  | 0.276 | 0.675 | - |  |
| **AP+EM** | 0.353 | 0.947 | 0.506 | - |  | 0.728 | **0.028*** | 0.895 | - |  | 0.319 | 0.606 | 0.922 | - |
| **5d** |  |  |  |  |  |  |  |  |  |  |  |  |  |  |
| **CON** | - |  |  |  |  | - |  |  |  |  | - |  |  |  |
| **HUA** | 0.070 | - |  |  |  | **0.001*** | - |  |  |  | 0.081 | - |  |  |
| **AP** | 0.947 | 0.079 | - |  |  | 0.111 | **0.037*** | - |  |  | 0.164 | 0.699 | - |  |
| **AP+EM** | 0.836 | 0.104 | 0.888 | - |  | 0.207 | **0.017*** | 0.718 | - |  | 0.351 | 0.388 | 0.629 | - |
| **7d** |  |  |  |  |  |  |  |  |  |  |  |  |  |  |
| **CON** | - |  |  |  |  | - |  |  |  |  | - |  |  |  |
| **HUA** | 0.351 | - |  |  |  | **<0.001*** | - |  |  |  | 0.105 | - |  |  |
| **AP** | 0.331 | 0.065 | - |  |  | 0.061 | **0.027*** | - |  |  | 0.326 | 0.497 | - |  |
| **AP+EM** | 0.672 | 0.182 | 0.577 | - |  | 0.224 | **0.005*** | 0.472 | - |  | 0.604 | 0.255 | 0.636 | - |
| **9d** |  |  |  |  |  |  |  |  |  |  |  |  |  |  |
| **CON** | - |  |  |  |  | - |  |  |  |  | - |  |  |  |
| **HUA** | 0.921 | - |  |  |  | **<0.001*** | - |  |  |  | 0.081 | - |  |  |
| **AP** | 0.142 | 0.119 | - |  |  | **0.032*** | **0.017*** | - |  |  | 0.224 | 0.565 | - |  |
| **AP+EM** | 0.676 | 0.749 | 0.065 | - |  | 0.103 | **0.004*** | 0.559 | - |  | 0.757 | 0.143 | 0.357 | - |

Note: Bold characteristic and *, significant difference between groups. CO, cortex; OS, outer stripe of the outer medulla; IS, inner stripe of the outer medulla; MK, mean kurtosis; MD, mean diffusivity; FA, fractional anisotropy; CON, control; HUA, hyperuricemia; AP, allopurinol; AP+EM, allopurinol+empagliflozin; base, basement.

| **Supplementary table 4 LSD as a post hoc test of one-way ANOVA for serum index of the four groups at different time points (*P*)** | | | | | | | | | | | | | | |
| --- | --- | --- | --- | --- | --- | --- | --- | --- | --- | --- | --- | --- | --- | --- |
|  | **uric acid** | | | |  | **serum creatinine** | | | |  | **blood urea nitrogen** | | | |
|  | **CON** | **HUA** | **AP** | **AP+EM** |  | **CON** | **HUA** | **AP** | **AP+EM** |  | **CON** | **HUA** | **AP** | **AP+EM** |
| **base** |  |  |  |  |  |  |  |  |  |  |  |  |  |  |
| **CON** | - |  |  |  |  | - |  |  |  |  | - |  |  |  |
| **HUA** | 0.809 | - |  |  |  | 0.720 | - |  |  |  | 0.715 | - |  |  |
| **AP** | 0.366 | 0.262 | - |  |  | 0.982 | 0.744 | - |  |  | 0.175 | 0.099 | - |  |
| **AP+EM** | 0.897 | 0.712 | 0.434 | - |  | 0.165 | 0.276 | 0.171 | - |  | 0.697 | 0.980 | 0.095 | - |
| **1d** |  |  |  |  |  |  |  |  |  |  |  |  |  |  |
| **CON** | - |  |  |  |  | - |  |  |  |  | - |  |  |  |
| **HUA** | **0.001*** | - |  |  |  | 0.997 | - |  |  |  | 0.531 | - |  |  |
| **AP** | **0.001*** | 0.821 | - |  |  | 0.613 | 0.616 | - |  |  | 0.601 | 0.265 | - |  |
| **AP+EM** | **0.001*** | 0.673 | 0.843 | - |  | 0.103 | 0.104 | 0.226 | - |  | 0.307 | 0.119 | 0.601 | - |
| **5d** |  |  |  |  |  |  |  |  |  |  |  |  |  |  |
| **CON** | - |  |  |  |  | - |  |  |  |  | - |  |  |  |
| **HUA** | **＜0.001*** | - |  |  |  | 0.577 | - |  |  |  | 0.489 | - |  |  |
| **AP** | **＜0.001*** | **0.031*** | - |  |  | 0.899 | 0.665 | - |  |  | 0.675 | 0.791 | - |  |
| **AP+EM** | **＜0.001*** | **0.016*** | 0.673 | - |  | 0.588 | 0.285 | 0.506 | - |  | 0.312 | 0.111 | 0.168 | - |
| **9d** |  |  |  |  |  |  |  |  |  |  |  |  |  |  |
| **CON** | - |  |  |  |  | - |  |  |  |  | - |  |  |  |
| **HUA** | **＜0.001*** | **-** |  |  |  | 0.907 | - |  |  |  | 0.708 | - |  |  |
| **AP** | **＜0.001*** | **0.020*** | - |  |  | 0.750 | 0.665 | - |  |  | 0.605 | 0.885 | - |  |
| **AP+EM** | **＜0.001*** | **0.012*** | 0.729 | - |  | 0.768 | 0.858 | 0.544 | - |  | 0.102 | 0.183 | 0.227 | - |

Note: Bold characteristic and *, significant difference between groups. CON, control; HUA, hyperuricemia; AP, allopurinol; AP+EM, allopurinol+empagliflozin; base, basement.

| **Supplementary table 5 LSD as a post hoc test of one-way ANOVA for the MOD of Masson trichrome staining of different groups at different time points (*P*)** | | | | | | |  |
| --- | --- | --- | --- | --- | --- | --- | --- |
|  |  |  |  |  |  |  |  |
|  | **OS** | | | **IS** | | |  |
| **1d** |  |  |  |  |  |  |  |
|  | **HUA** | **AP** | **AP+EM** | **HUA** | **AP** | **AP+EM** |  |
| **HUA** | - |  |  | - |  |  |  |
| **AP** | 0.251 | - |  | 0.275 | - |  |  |
| **AP+EM** | **0.049*** | 0.279 | - | 0.083 | 0.415 | - |  |
| **5d** |  |  |  |  |  |  |  |
| **HUA** | - |  |  | - |  |  |  |
| **AP** | 0.125 | - |  | 0.320 | - |  |  |
| **AP+EM** | **0.049*** | 0.524 | - | **0.008*** | **0.030*** | - |  |
| **9d** |  |  |  |  |  |  |  |
| **HUA** | **-** |  |  | - |  |  |  |
| **AP** | 0.085 | - |  | **0.035*** | - |  |  |
| **AP+EM** | **0.021*** | 0.344 | - | **0.001*** | **0.011*** | - |  |

Note: Bold characteristic and *, significant difference between groups.

OS, outer stripe of the outer medulla; IS, inner stripe of the outer medulla;

HUA, hyperuricemia; AP, allopurinol; AP+EM, allopurinol+empagliflozin;

MOD, mean optical density.

.

| **Supplementary table 6 LSD as a post hoc test of one-way ANOVA for the MOD of NF-κB immunostaining of different groups at different time points (*P*)** | | | | | | |  |
| --- | --- | --- | --- | --- | --- | --- | --- |
|  |  |  |  |  |  |  |  |
|  | **OS** | | | **IS** | | |  |
| **1d** |  |  |  |  |  |  |  |
|  | **HUA** | **AP** | **AP+EM** | **HUA** | **AP** | **AP+EM** |  |
| **HUA** | - |  |  | - |  |  |  |
| **AP** | 0.546 | - |  | 0.056 | - |  |  |
| **AP+EM** | 0.134 | 0.316 | - | **0.022*** | 0.512 | - |  |
| **5d** |  |  |  |  |  |  |  |
| **HUA** | - |  |  | - |  |  |  |
| **AP** | 0.129 | - |  | 0.147 | - |  |  |
| **AP+EM** | **0.018*** | 0.196 | - | **0.012*** | 0.105 | - |  |
| **9d** |  |  |  |  |  |  |  |
| **HUA** | **-** |  |  | - |  |  |  |
| **AP** | **0.003*** | - |  | 0.188 | - |  |  |
| **AP+EM** | **0.001*** | 0.192 | - | **0.017*** | 0.127 | - |  |

Note: Bold characteristic and *, significant difference between groups.

OS, outer stripe of the outer medulla; IS, inner stripe of the outer medulla;

HUA, hyperuricemia; AP, allopurinol; AP+EM, allopurinol+empagliflozin;

MOD, mean optical density.
